# Ruthenacarborane–Phenanthroline Derivatives as Potential Metallodrugs

**DOI:** 10.3390/molecules25102322

**Published:** 2020-05-15

**Authors:** Martin Kellert, Imola Sárosi, Rajathees Rajaratnam, Eric Meggers, Peter Lönnecke, Evamarie Hey-Hawkins

**Affiliations:** 1 Institute of Inorganic Chemistry, Faculty of Chemistry and Mineralogy, Leipzig University, Johannisallee 29, 04103 Leipzig, Germany; martin.kellert@uni-leipzig.de (M.K.); sarosi.imola@gmail.com (I.S.); loenneck@uni-leipzig.de (P.L.); 2 Fachbereich Chemie, Philipps-Universität Marburg, Hans-Meerwein Straße 4, 35043 Marburg, Germany; Rajathees@Rajaratnam.de (R.R.); meggers@chemie.uni-marburg.de (E.M.)

**Keywords:** dicarbollide, ruthenium, metallodrug, kinase inhibitor

## Abstract

Ruthenium-based complexes have received much interest as potential metallodrugs. In this work, four Ru^II^ complexes bearing a dicarbollide moiety, a carbonyl ligand, and a phenanthroline-based ligand were synthesized and characterized, including single crystal diffraction analysis of compounds **2**, **4**, and **5** and an observed side product **SP1**. Complexes **2**–**5** are air and moisture stable under ambient conditions. They show excellent solubility in organic solvents, but low solubility in water.

## 1. Introduction

Metal-containing compounds are of increasing interest for applications in medicinal chemistry due to their diverse coordination geometries, unusual reactivities, and useful physicochemical properties [1,2,3]. Ruthenium shows a low general toxicity [4] and is an excellent metal for this approach. Ru^II^ is usually coordinated in an octahedral or pseudo-octahedral half-sandwich fashion and forms quite stable coordinative or covalent Ru–ligand bonds, which affects cellular metabolism. Furthermore, the reactivity of ruthenium ions is well-known; thus, reactions are predictable and facilitate drug design [5,6,7,8]. To date, predominantly ruthenium half-sandwich complexes have been developed as potential anti-cancer agents, antiproliferative drugs, antibiotics, and immunosuppressants [9,10,11,12,13,14].

**Figure 1:**
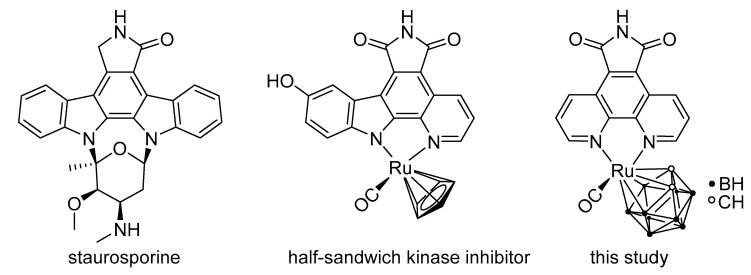
Staurosporine, a ruthenium half-sandwich staurosporine mimic (DW12), and the ruthenacarborane complex synthesized and investigated in this study [17].

Meggers et al., recently reported an interesting class of organometallic protein kinase inhibitors which were inspired by the alkaloid staurosporine (Figure 1) [4,15,16]. For example, the ruthenium half-sandwich complex DW12 is a potent inhibitor of glycogen synthase kinase 3 (GSK-3), whereas staurosporine constitutes a very unselective inhibitor of a large number of protein kinases [5,16,17,18]. In staurosporine, the important groups for the interaction with protein kinase are the lactam unit, the indolocarbazole heterocycle, and the carbohydrate moiety. In DW12, the ruthenium serves as a purely structural center and enables a geometry, which cannot be easily achieved with purely organic molecules. Thus, the NH group of the maleimide moiety forms a hydrogen bond with the amide carbonyl of Asp133 in the adenosine triphosphate (ATP) binding site of GSK-3. Additionally, one carbonyl group of the maleimide moiety interacts with the NH group of Val135, and the second carbonyl of the same moiety forms a water-mediated hydrogen bond with Asp200. The indole OH group interacts with the carbonyl amide group of Val135. One special feature of DW12 is the presence of the CO ligand, which exhibits a clearly reduced dipolar character due to the interaction with the transition metal ruthenium. Displaying this behavior, the CO ligand is able to undergo an unusual interaction mode with the glycine-rich loop of the ATP binding site. In that way, DW12 achieves a geometry, which seems to be optimal for the ATP binding site of GSK-3, rendering DW12 a more potent inhibitor for GSK-3 than staurosporine. 

In order to design novel potential drugs, bioisosteric replacement has become a wide-spread approach [11,14,19,20,21,22,23]. Thus, the development of drugs in which the carborane moiety mimics a phenyl group or is the pharmacophore itself is actively studied [23,24,25,26,27,28,29]. Applications of such carborane-containing drugs are for example cancer therapeutics and enzyme inhibitors [11,14,19,20,22,23,30,31,32,33,34,35,36,37]. Due to its isolobal relationship with the cyclopentadienyl ligand (Cp^−^) the dicarbollide anion (*nido*-carborate(2−), C_2_B_9_H_11_^2−^, Cb^2−^) is a suitable replacement as ligand for transition metals [38,39]. However, distinct activities and reactivities of the respective metal complexes, bearing the Cb^2−^ or the Cp^−^, are observable and caused by the different charge, size, symmetry, and hybridization of the orbitals of the respective ligands [40]. Furthermore, the dicarbollide moiety is highly hydrophobic and could enhance the transport of the corresponding metallodrug across cellular membranes [19,20,22]. Additionally, these clusters are metabolically stable, which renders them robust compounds in biological media [11,23]. Furthermore, the possible regioselective introduction of specific substituents at either the carbon or the boron vertices of the cluster enables customization of the structure and, therefore, the activity of the metallodrug [19,21].

Former studies have shown the importance of the heteroaromatic bidentate pyridocarbazole and CO ligand in DW12 and related complexes for mimicking staurosporine binding in the ATP binding site [5,17,18,41]. Therefore, the presence of a carbonyl ligand and an aromatic moiety are very important features. 

In this work, we report the combination of Cb^2−^ with Ru^II^(CO)–phenanthroline derivatives as mimics for DW12. To our knowledge, this approach to combine the scaffold of an active ruthenium-based protein kinase inhibitor with a dicarbollide moiety has not been pursued before. In DW12, the Cp^−^ ring points away from the ATP binding site towards the aqueous solvent. Thus, there should be sufficient space available in this part of the active site to accommodate larger moieties [42]. Replacing the Cp^−^ ligand with a much bulkier, hydrophobic Cb^2−^ ligand would allow additional van der Waals interactions to be formed with this part of the active site and to profit from the hydrophobic effect which often increases the potency of enzyme inhibitors. Due to the replacement of the cyclopentadienyl ligand with a dicarbollide ligand, the anionic *N*,*N* ligand in DW12 must be substituted with a neutral one. As the maleimide moiety in the *N*,*N* ligand in DW12 is involved in various hydrogen bond interactions and thus plays an essential role in binding to the ATP binding site of GSK-3, the design of the novel dicarbollide-containing complex should also employ this motif. Therefore, the neutral 5*H*-pyrrolo[3,4-f][1,10]phenanthroline-5,7(6*H*)-dione, which is similar to the anionic 9-hydroxy-5*H*-12λ^2^-pyrido[2,3-*a*]pyrrolo[3,4-*c*]carbazole-5,7(6*H*)-dione ligand (pyridocarbazole) in DW12, was used as ligand. In combination with the much more hydrophobic and bulkier Cb^2−^ ligand, this should result in increased activity of the respective complex. 

## 2. Results and Discussion

### 2.1. Ligand and Precursor Syntheses

**Scheme 1:**
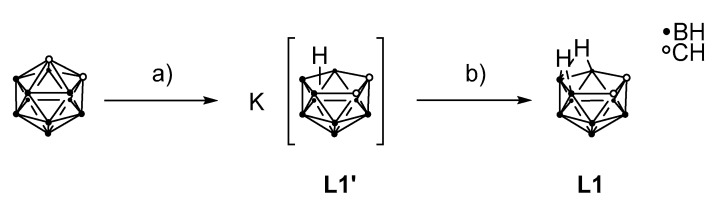
Two-step synthesis of 7,8-dicarba-*nido*-undecaborane(13) (**L1**). a) MeOH, KOH, 80 °C, 18 h; b) C_6_H_6_, H_3_PO_4_, rt, 17 h. Yield over two steps: 70% [43].

The precursor 7,8-dicarba-*nido*-undecaborane(13) (**L1**) for the dicarbollide moiety was synthesized according to the literature (Scheme 1) [43]. Details about the synthetic procedure are given in the electronic supplementary information.

**Figure 2:**
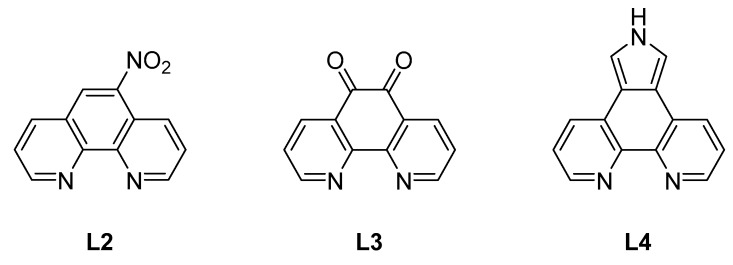
Phenanthroline-based ligands **L2**–**L4**.

**Scheme 2:**
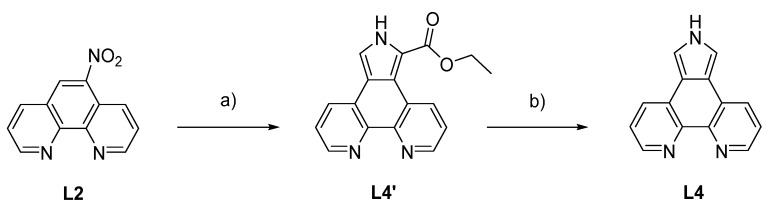
Preparation of **L4** starting from 5-nitro-1,10-phenanthroline (**L2**) with 1,10-phenanthrolinopyrrole ethyl ester (**L4’**) as intermediate. a) THF, DBU, ethyl isocyanoacetate, rt, 20 h; b) EtOH, 0.2 M NaOH, 110 °C, 8 h. Yield over two steps: 54%; DBU: 1,8-diazabicyclo[5.4.0]undec-7-ene [49].

The phenanthroline derivatives (Figure 2) that were employed in complexation reactions were prepared according to the literature. Even though 5-nitro-1,10-phenanthroline (**L2**) can be synthesized in excellent yields, it was obtained commercially because of the harsh conditions employed in the synthesis [44]. 1,10-Phenanthroline-5,6-dione (**L3**) was prepared according to the literature [45,46], and 1,10-phenanthrolinopyrrole (**L4**) was formed in a Barton-Zard reaction from 5-nitro-1,10-phenanthroline (**L2**) and ethyl isocyanoacetate under basic conditions followed by hydrolysis of the ester **L4’** (Scheme 2) [47,48,49,50,51,52,53,54] The respective synthetic procedure for **L4** is given in the electronic supplementary information.

**Scheme 3:**
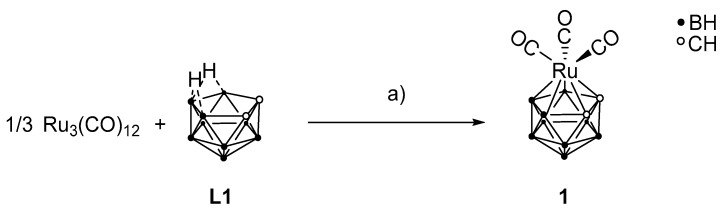
Preparation of **1** with triruthenium dodecacarbonyl and **L1**. a) C_6_H_6_, 90 °C, 7 h, 60% [55].

The precursor molecule for further reactions, [3-(CO)_3_-*closo*-3,1,2-RuC_2_B_9_H_11_] (**1**), was prepared in a redox reaction from triruthenium dodecacarbonyl and **L1** (Scheme 3) [55,56,57]. The respective synthetic procedure of **1** is given in the electronic supplementary information. Studies showed that the dicarbollide ligand is a much stronger ligand than the Cp ligand [11,39].

### 2.2. Synthesis of Ruthenium(II) Complexes

**Scheme 4:**
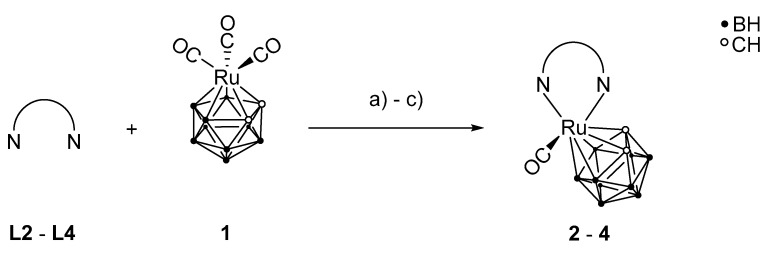
Preparation of compounds **2**–**4** from the phenanthroline ligands **L2**–**L4** and the ruthenium(II) complex **1**. a) MeCN, trimethylamine *N*-oxide, rt, 17 h, 13% **2**; b) MeCN, trimethylamine *N*-oxide, rt, 84 h, 73% **3**; c) MeCN, trimethylamine *N*-oxide, rt, 48 h, 46% **4**.

The complexes containing phenanthroline derivatives as ligands were prepared via a ligand exchange reaction following a procedure of Jellis and co-workers for other chelating *N*-donor ligands (Scheme 4) [58]. In this type of reaction, two carbonyl ligands are oxidized to CO_2_ with stoichiometric amounts of trimethylamine *N*-oxide [59] to facilitate coordination of one bidentate phenanthroline derivative. For complex **2**, both enantiomers *R* and *S* were obtained (Ru is the chiral center). No further investigations were carried out to determine the ratio of the two enantiomers, which is assumed to be close to 1:1.

Complexes **2**–**4** were characterized via NMR, IR, MS, and, in the case of compounds **2** and **4**, via single crystal X-ray diffraction. In the ^1^H-NMR spectrum, compound **3** exhibits three doublets of doublets in the aromatic region as expected for the dione ligand **L3**. In the infrared spectrum only one absorption band for a C≡O stretching vibration was observed for **2**–**4** for the single remaining CO ligand. Additionally, in **2**, two absorption bands for the symmetric and asymmetric stretching vibration of the NO_2_ group were observed. In the negative ESI-MS spectrum, compound **3** is observable with one additional bromide ion ([M + Br]^−^, *m*/*z* = 552). No suitable single crystals could be obtained for complex **3**. The bifunctional ligand **L3** can exhibit several different binding modes: η^2^-coordination with both nitrogen or both oxygen atoms. In **3**, coordination via the nitrogen atoms is assumed, as Ru^2+^ is a soft Lewis acid [60,61]. It is also possible for **L3** to act as a bridging ligand to form complexes with different or additional metal centers. In our case the formation of chain-like oligomers formed by linked complex fragments of **3** are not very likely due to the chosen synthetic procedure.

**Figure 3:**
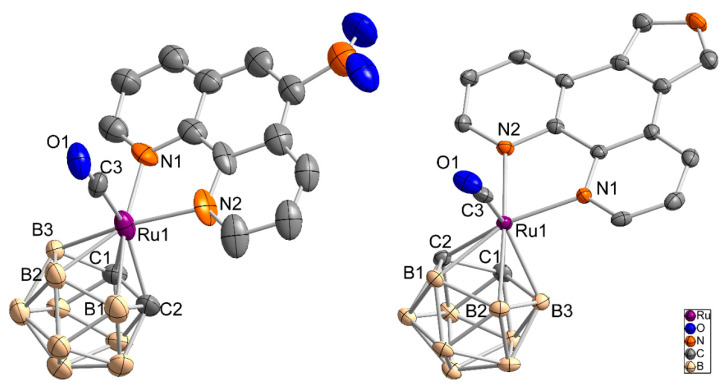
Molecule structure of [3-(CO)-3,3-(**L2**-κ^2^*N,N*)-*closo*-3,1,2-RuC_2_B_9_H_11_] (**2**) (**left**, only the *S* enantiomer is shown) and [3-(CO)-3,3-(**L4**-κ^2^*N,N*)-*closo*-3,1,2-RuC_2_B_9_H_11_] (**4**) (**right**) as an ellipsoid-stick model with thermal ellipsoids at 50% probability level. Solvent molecules and hydrogen atoms are omitted for clarity.

Crystals suitable for single crystal X-ray crystallography were obtained for compounds **2** and **4**. Compound **2** crystallizes from a mixture of dichloromethane and *n*-hexane as orange-red, plate-like crystals in the monoclinic space group *P*2_1_/*n* with one additional DCM molecule in the asymmetric unit. The solved structure shows a *wR*_2_ value of 9.9% (*R*_1_: 17.8%), which is caused by the low quality of the crystals. Nonetheless, the identity of compound **2** is unambiguous (Figure 3, left). Complex **4** crystallizes as yellow plates from a dichloromethane/*n*-hexane mixture with two independent molecules in the asymmetric unit (Figure 3, right, only one molecule is shown). 

Bond lengths and bond angles of **2** and **4** are given in Table 1.

**Scheme 5:**
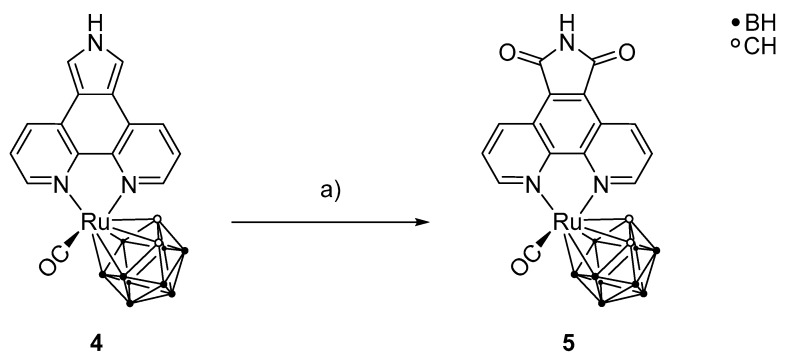
Synthesis of [3-(CO)-3,3-{1′,10′-NC_5_H_3_(C(CO)(NH)(CO)C)NC_5_H_3_-κ^2^*N,N*}-*closo*-3,1,2-RuC_2_B_9_H_11_] (**5**) from **4** and *meta*-chloroperoxybenzoic acid (*m*-CPBA). a) MeCN, *m*-CPBA, 82 °C, 84 h, 10%.

After the successful preparation of **4**, a selective oxidation of positions 5 and 7 of the phenanthrolinopyrrole moiety was carried out to prepare [3-(CO)-3,3-{1′,10′-NC_5_H_3_(C(CO)(NH)(CO)C)NC_5_H_3_-κ^2^*N,N*}-*closo*-3,1,2-RuC_2_B_9_H_11_] (**5**) (Scheme 5). For this reaction, excess of *meta*-chloroperoxybenzoic acid (*m*-CPBA) was used. For stronger oxidizing agents, a polymerization of the pyrrole moiety is observed, and milder oxidizing agents lead to lactam scaffolds only and not to the desired maleimide groups [62,63]. Monitoring the reaction using thin layer chromatography showed that it is necessary to add the oxidizing agent successively in small portions during the reaction period. It was observed that only the *ortho* positions of the pyrrole moiety were oxidized, whereas the rest of the complex was unaffected.

**Figure 4:**
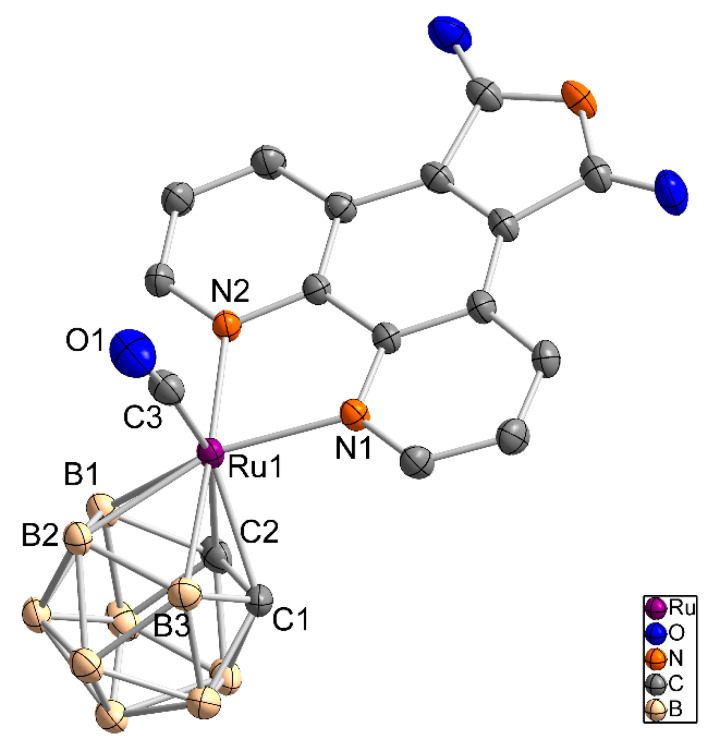
Molecule structure of [3-(CO)-3,3-{1′,10′-NC_5_H_3_(C(CO)(NH)(CO)C)NC_5_H_3_-κ^2^*N,N*}-*closo*-3,1,2-RuC_2_B_9_H_11_] (**5**) as an ellipsoid-stick model with thermal ellipsoids at 50% probability level. Solvent molecules and hydrogen atoms are omitted for clarity.

After column chromatography and recovery of some starting material, the desired complex **5** was isolated in 10% yield as a red, crystalline powder and characterized. In the ^1^H-NMR spectrum, a low-field shift for the cluster CH and the aromatic CH groups in comparison to **4** was observed. The multiplicity pattern of the aromatic protons indicates an *ortho* substitution of the pyrrole moiety. Two signals are observed in the negative-mode mass spectrum, namely [M − H^+^]^−^ and [M − CO − H^+^]^−^. The IR spectrum shows the presence of only one CO ligand as well as for the previous complexes. Compound **5** crystallizes from acetonitrile as deep red prisms in the triclinic space group *P*$\bar{1}$ with three acetonitrile molecules in the asymmetric unit (Figure 4). Bond lengths and bond angles of **5** are given in Table 1.

### 2.3. Biological Studies

Since the target molecule **5** was designed as a mimic of (*R*)-DW12, protein kinase Pim1 inhibition studies were carried out but were inconclusive, most likely due to a low solubility of **5** in buffer solutions caused by the high hydrophobicity of the dicarbollide moiety. Additional information about the protein kinase inhibition studies is given in the electronic supplementary information.

As mentioned in the introduction, carborane or metallacarborane derivatives are being studied as drugs or enzyme inhibitors [11,14,19,21,23,27,28,30,35]. Due to the presence of the hydrophobic carborane moiety, lack of solubility in aqueous media is often observed, hampering biological studies. Improved water-solubility was achieved by employing charged species with enhanced solubility [11,14,31,35] or incorporating hydrophilic side chains [23]. A novel approach, developed by Hey-Hawkins et al., overcomes the problematic solubility behavior of metallacarborane complexes by employing bovine serum albumin (BSA) [37]; however, it was also observed that BSA can influence the activity of the solubilized drug in specific enzyme inhibition assays.

## 3. Materials and Methods

All syntheses were carried out using the Schlenk technique and nitrogen as inert gas. Triruthenium dodecacarbonyl, 1,2-dicarba-*closo*-dodecaborane(12), 5-nitro-1,10-phenanthroline, ethyl isocyanoacetate, 1,8-diazabicyclo[5.4.0]undec-7-ene, trimethylamine *N*-oxide and *meta*-chloroperoxybenzoic acid are commercially available. Trimethylamine *N*-oxide was dried and purified by sublimation; *meta*-chloroperoxybenzoic acid was purified by washing with phosphate buffer and drying under reduced pressure. 7,8-Dicarba-*nido*-undecaborane(13) (**L1**) [43], 1,10-phenanthrolinopyrrole (**L2**) [49], [3-(CO)_3_-*closo*-3,1,2-RuC_2_B_9_H_11_] (**1**) [55], and 1,10-phenanthrolino-5,6-dione (**L5**) [45,46] were synthesized according to the literature. The oxidation-sensitive compound 7,8-dicarba-*nido*-undecaborane(13) (L**1**) was stored under nitrogen at −55 °C. Ethyl isocyanoacetate was stored under nitrogen atmosphere and *meta*-chloroperoxybenzoic acid under normal atmosphere at 4 °C. All other used chemicals were stored under nitrogen atmosphere at ambient temperatures. All solvents, except acetonitrile, benzene, cyclohexane, and methanol, which were dried over calcium hydride, sodium, or calcium oxide, respectively, were taken from the solvent purification system MB SPS-800 (by MBraun). Ethanol was used as a mixture with water. Petrol ether (40–60 °C) for column chromatography was used as provided.

NMR spectra were measured with an ADVANCE DRX 400 spectrometer from Bruker. The spectrometer frequency for ^1^H and ^11^B are 400.13 MHz and 128.38 Hz, respectively. As an internal standard, tetramethylsilane was used for ^1^H-NMR and the Ξ scale was used for ^11^B-NMR spectroscopy. Data were interpreted with MestReNova [64]. The numbering scheme of all isolated compounds is given in the electronic supplementary information. Positive- or negative-mode low-resolution electrospray ionization mass spectra (ESI-MS) were recorded with an ESI ESQUIRE 3000 PLUS spectrometer with an IonTrap analyzer from Bruker Daltonics. For these measurements, dichloromethane, acetonitrile, methanol, or a mixture of these solvents were used. Infrared spectra were recorded with a Spectrum 2000 IR spectrometer from PerkinElmer in the range of 400 to 4000 cm^−1^. All samples were prepared as KBr pellets. The determination of the single crystal structures was carried out with a Gemini-S diffractometer from Oxford Diffraction using MoK_α_ radiation (λ = 71.073 pm). The visualization of the structures was carried out with Diamond [65]. Additional crystallographic data are given in the electronic supplementary information. For column chromatography, silica gel 60 Å from the company Acros was used. The particle size was in the range of 0.035 to 0.070 mm. The solvents for semi-inert chromatography were obtained from the solvent purification device MB SPS-800. Thin layer chromatography (TLC) was used to monitor reaction processes using glass plates coated with silica gel 60 F_254_ from Merck. Carborane-containing spots were stained with a 5% solution of palladium(II) chloride in methanol.

*[3-(CO)-3,3-(**L2**-κ^2^N,N)-closo-3,1,2-RuC_2_B_9_H_11_]* (**2**): 0.10 g (0.32 mmol, 1.00 eq.) **1** were placed in a 250 mL round bottom flask and dissolved in 25 mL acetonitrile. With stirring, a solution of 0.05 g (0.64 mmol, 2.00 eq.) trimethylamine *N*-oxide in 10 mL acetonitrile was added. The mixture was stirred for 10 min. Subsequently, 0.07 g (0.31 mmol, 0.97 eq.) 5-nitro-1,10-phenanthroline (**L2**), dissolved in 20 mL acetonitrile, were added dropwise to the mixture. The reaction mixture was stirred for 17 h at rt. After the reaction was finished (monitored using TLC), the mixture was filtered and the filtrate was dried under reduced pressure. The product was purified using column chromatography (dichloromethane; *R_f_* = 0.60). **2** (0.02 g, 0.04 mmol, 13%) was obtained as an orange-red crystalline solid. ^1^H-NMR (400 MHz, CD_3_CN): δ = 1.00–2.80 (br, 9 H, 9× BH), 3.39 (s, br, 2 H, 2× CH^1^), 8.05 (m, 2 H, CH^3^, CH^7^), 8.88 (d, ^3^*J*_HH_ = 8.2 Hz, 1 H, CH^6^), 9.10 (s, 1 H, CH^5^), 9.23 (d, ^3^*J*_HH_ = 8.6 Hz, 1 H, CH^4^), 9.49 (d, ^3^*J*_HH_ = 5.2 Hz, 1 H, CH^8^), 9.53 ppm (d, ^3^*J*_HH_ = 5.3 Hz, 1 H, CH^2^); ^11^B{^1^H}-NMR (128 MHz, CD_3_CN): δ = −22.2 (s, 1 B, BH), −21.4 (s, 2 B, BH), −9.8 (s, 2 B, BH), −8.9 (s, 2 B, BH), −6.8 (s, 1 B, BH), −2.0 ppm (s, 1 B, BH); ^11^B-NMR (128 MHz, CD_3_CN): δ = −21.8 (m, br, 3 B, BH), −9.3 (m, br, 4 B, BH), −6.8 (d, ^1^*J*_BH_ = 139 Hz, 1 B, BH), −2.0 ppm (d, ^1^*J*_BH_ = 135 Hz, 1 B, BH); IR (KBr): $\tilde{\nu }$ = 2524 (s, νBH-sp^3^), 1970 (s, νCO-sp), 1535 (m, ν_asym._NO_2_), 1514 (m, νCN-sp^2^), 1342 cm^−1^ (m, ν_sym._NO_2_); MS (ESI, neg.): found: *m*/*z* (%): 549 (100) [M + NO_3_]^−^; calcd: *m*/*z*: 549 [M + NO_3_]^−^.
*[3-(CO)-3,3-(**L3**-κ^2^N,N)-closo-3,1,2-RuC_2_B_9_H_11_]* (**3**): 0.10 g (0.32 mmol, 1.00 eq.) **1** were placed in a 250 mL round bottom flask and dissolved in 25 mL acetonitrile. Subsequently, 0.05 g (0.64 mmol, 2.00 eq.) trimethylamine *N*-oxide, dissolved in 10 mL acetonitrile, were added. The mixture was stirred for 10 min at rt. Then, 0.07 g (0.33 mmol, 1.03 eq.) 1,10-phenanthroline-5,6-dione (**L3**), dissolved in 15 mL acetonitrile, were added dropwise to the mixture. The reaction mixture was stirred for 84 h at rt. After the reaction was complete (monitored using TLC), the resulting precipitate was filtered off and the filtrate was concentrated under reduced pressure. The product was purified using column chromatography (dichloromethane; *R_f_* = 0.17). Yield of **3**: 0.11 g (0.23 mmol, 73%), orange solid. ^1^H-NMR (400 MHz, CD_3_CN): δ = 1.00–3.90 (br, 9 H, 9 × BH), 3.37 (s, br, 2 H, 2× CH^1^), 7.82 (dd, ^3^*J*_HH_ = 7.9 Hz, ^3^*J*_HH_ = 5.6 Hz, 2 H, 2× CH^3^), 8.62 (dd, ^3^*J*_HH_ = 7.9 Hz, ^4^*J*_HH_ = 1.4 Hz, 2 H, 2× CH^4^), 9.26 ppm (dd, ^3^*J*_HH_ = 5.6 Hz, ^4^*J*_HH_ = 1.4 Hz, 2 H, 2× CH^2^); ^11^B{^1^H}-NMR (128 MHz, CD_3_CN): δ = −21.9 (s, br, 3 B, BH), −9.6 (s, br, 4 B, BH), −7.8 (s, 1 B, BH), −1.4 ppm (s, 1 B, BH); ^11^B-NMR (128 MHz, CD_3_CN): δ = −21.9 (d, ^1^*J*_BH_ = 143 Hz, 3 B, BH), −9.6 (d, ^1^*J*_BH_ = 142 Hz, 4 B, BH), −7.8 (d, ^1^*J*_BH_ = 144 Hz, 1 B, BH), −1.4 ppm (d, ^1^*J*_BH_ = 151 Hz, 1 B, BH); IR (KBr): $\tilde{\nu }$ = 2507 (m, νBH-sp^3^), 1984 (m, νCO-sp), 1702 (m, νCO-sp^2^), 1691 (m, νCN-sp^2^), 802 cm^−1^ (s, 1,2,3-trisubstituted aromatic ring); MS (ESI, neg.): found: *m*/*z* (%): 552 (67) [M + Br]^−^; calcd: *m*/*z*: 552 [M + Br]^−^.
*[3-(CO)-3,3-(**L4**-κ^2^N,N)-closo-3,1,2-RuC_2_B_9_H_11_]* (**4**): 0.20 g (0.63 mmol, 1.00 eq.) **1** were placed in a 250 mL round bottom flask and dissolved in 25 mL acetonitrile. To this solution, 0.10 g (1.26 mmol, 2.00 eq.) trimethylamine *N*-oxide, dissolved in 10 mL acetonitrile, were added dropwise. The mixture was stirred for 10 min at rt. Then, 0.28 g (1.28 mmol, 2.03 eq.) 1,10-phenanthrolinopyrrole (**L4**) were added in one portion and the reaction mixture was stirred for 48 h at room temperature. After the reaction was completed (monitored using TLC), the resulting precipitate was filtered off and the filtrate was concentrated under reduced pressure. The product was purified using column chromatography (dichloromethane; *R_f_* = 0.33). Yield of **4**: 0.14 g (0.29 mmol, 46%), yellow crystalline solid. ^1^H-NMR (400 MHz, CD_3_CN): δ = 0.70–2.80 (br, 9 H, 9× BH), 3.28 (s, br, 2 H, 2× CH^1^), 7.71 (dd, ^3^*J*_HH_ = 8.1 Hz, ^3^*J*_HH_ = 5.4 Hz, 2 H, 2× CH^3^), 7.92 (d, ^3^*J*_HH_ = 2.8 Hz, 2 H, 2× CH^5^), 8.67 (d, ^3^*J*_HH_ = 7.7 Hz, 2 H, 2× CH^4^), 9.03 (d, ^3^*J*_HH_ = 5.1 Hz, 2 H, 2× CH^2^), 10.70 ppm (s, br, 1 H, NH^6^); ^11^B{^1^H}-NMR (128 MHz, CD_3_CN): δ = −21.7 (s, br, 3 B, BH), −10.1 (s, 2 B, BH), −9.0 (s, 2 B, BH), −7.2 (s, br, 1 B, BH), −2.7 ppm (s, br, 1 B, BH); ^11^B-NMR (128 MHz, CD_3_CN): δ = −21.8 (m, 3 B, BH), −8.8 (m, br, 5 B, BH), −2.7 ppm (d, ^1^*J*_BH_ = 139 Hz, 1 B, BH); IR (KBr): $\tilde{\nu }$ = 2523 (m, νBH-sp^3^), 1958(s, νCO-sp), 1639 (w, νCN-sp^2^), 1600 (w, νCC-sp^2^), 803 cm^−1^ (m, 1,2,3-trisubstituted aromatic ring); MS (ESI, neg.): found: *m*/*z* (%): 480 (100) [M − H]^−^; calcd: *m*/*z*: 480 [M − H]^−^.
*[3-(CO)-3,3-{1′,10′-NC_5_H_3_(C(CO)(NH)(CO)C)NC_5_H_3_-κ^2^N,N}-closo-3,1,2-RuC_2_B_9_H_11_]* (**5**): 0.31 g (0.65 mmol, 1.00 eq.) **4** were placed in a 250 mL round bottom flask and dissolved in 30 mL acetonitrile. Subsequently, 0.45 g (2.61 mmol, 4.01 eq.) *m-*CPBA, dissolved in 10 mL acetonitrile, were added under stirring at rt. The reaction mixture was heated under reflux for 84 h. During this time, two additional portions of 0.28 g (1.62 mmol, 2.49 eq.) *m-*CPBA, dissolved in 10 mL acetonitrile, were added after 24 h and 48 h, respectively. After completion of the reaction (monitored using TLC), the mixture was cooled to rt; the resulting precipitate was filtered off and the filtrate was concentrated under reduced pressure. The product was purified using column chromatography (dichloromethane/acetonitrile, 10:1, (*v*/*v*); *R_f_* = 0.52). Yield of **5**: 0.03 g (0.06 mmol, 9%, corrected, after recovery of starting material **4**: 10%), deep red crystalline powder. In addition, 0.03 g (0.06 mmol) **4** were recovered. ^1^H-NMR (400 MHz, acetone-*d_6_*): δ = 0.82–3.01 (br, 9 H, 9× BH), 3.53 (s, br, 2 H, 2× CH^1^), 8.31 (dd, ^3^*J*_HH_ = 8.3 Hz, ^3^*J*_HH_ = 5.2 Hz, 2 H, 2× CH^3^), 9.59 (dd, ^3^*J*_HH_ = 8.3 Hz, ^3^*J*_HH_ = 1.4 Hz, 2 H, 2× CH^4^), 9.73 (dd, ^3^*J*_HH_ = 5.3 Hz, ^3^*J*_HH_ = 1.4 Hz, 2 H, 2× CH^2^), 10.74 ppm (s, br, 1 H, NH^5^); ^11^B{^1^H}-NMR (128 MHz, acetone-*d_6_*): δ = −21.7 (s, br, 3 B, BH), −9.3 (s, 2 B, BH), −7.5 (s, 3 B, BH), −1.3 ppm (s, 1 B, BH); ^11^B-NMR (128 MHz, acetone-*d_6_*): δ = −21.7 (d, ^1^*J*_BH_ = 157 Hz, 3 B, BH), −9.2 (d, ^1^*J*_BH_ = 162 Hz, 2 B, BH), −7.5 (d, ^1^*J*_BH_ = 150 Hz, 3 B, BH), −1.3 ppm (d, ^1^*J*_BH_ = 142 Hz, 1 B, BH); IR (KBr): $\tilde{\nu }$ = 2531 (s, νBH-sp^3^), 1956 (s, νCO-sp), 1727 (s, νCO-sp^2^), 1695 cm^−1^ (m, νCN-sp^2^); MS (ESI, neg.): found: *m*/*z* (%): 510 (100) [M − H]^−^, 482 (26) [M − CO − H]^−^; calcd: *m*/*z*: 510 [M − H]^−^, 482 [M − CO − H]^−^.

## 4. Conclusions

Four air stable ruthenium(II) half-sandwich complexes, **2**‒**5**, which contain a dicarbollide moiety, a carbonyl ligand, and a phenanthroline derivative, were prepared in moderate to good yields and fully characterized. The complexes were designed to mimic the overall shape and structure of the alkaloid staurosporine and the ruthenium half-sandwich complex DW12. Initial inhibition experiments with the ruthenium(II) complexes **4** and **5** against the protein kinase Pim1 were not conclusive, most likely due to a low solubility of **4** and **5** in buffer solutions caused by the high hydrophobicity of the dicarbollide moiety. Thus, although a high hydrophobicity is beneficial for the inhibition of a biological target molecule, a sufficient solubility in aqueous buffer solution must be warranted, which will need to be addressed in future work. 

## Figures and Tables

**Table 1:** Selected bond lengths (in pm) and angles (in °) in **2**, **4**, and **5** in comparison with [3-CO-3,3-(bipy-κ^2^*N,N*)-*closo*-3,1,2-RuC_2_B_9_H_11_] (**I**) (bipy = 2,2′-bipyridine) [58].

Atom Group	2	4 ^a^	5	I
Ru(1)–C(1)	226.5(9)	222.3(4) [225.0(4)]	221.3(2)	217.4(4)
Ru(1)–C(2)	222.7(9)	217.6(4) [223.4(49]	222.7(2)	222.4(4)
Ru(1)–B(1)	220(1)	222.4(4) [220.1(4)]	220.6(2)	227.9(4)
Ru(1)–B(2)	219(1))	227.3(4) [220.5(4)]	224.0(2)	227.7(4)
Ru(1)–B(3)	221.5(9)	223.4(4) [223.6(4)]	224.0(2)	220.6(5)
Ru(1)–C(3)	185(1)	185.6(4) [183.5(4)]	184.6(2)	186.6(4)
Ru(1)–N(1)	212.9(8)	210.5(3) [211.6(3)]	212.2(1)	209.3(3)
Ru(1)–N(2)	213.6(7)	212.4(3) [212.2(3)]	211.9(1)	213.5(3)
N(1)···N(2)	261(1)	262.5(5) [262.3(4)]	262.8(2)	261.0(5)
C(3)–O(1)	115(1)	114.7(4) [114.6(4)]	115.0(2)	115.4(5)
N(1)–Ru(1)–C(3)	90.9(4)	91.5(1) [94.9(2)]	93.9(1)	90.1(2)
N(2)–Ru(1)–C(3)	91.0(4)	95.5(1) [94.0(1)]	92.2(1)	92.0(1)
N(1)–Ru(1)–N(2)	75.5(3)	76.7(1) [76.5(1)]	76.6(1)	76.3(1)
Ru(1)–C(3)–O(1)	176.4(9)	173.4(3) [174.1(4)]	173.8(2)	175.3(4)

^a^ values of the second independent molecule of **4** are given in [ ].

## Supplementary Materials

The electronic supplementary information is available online including the numbering scheme of all isolated compounds, additional synthetic procedures for **L1**, **L4**, and **1,** the isolation of **SP1** and its spectroscopic data, crystallographic information of compounds **2**, **SP1**, **4**, and **5,** and information about the protein kinase inhibition studies.
