# Supplementary material for: Ruthenacarborane–Phenanthroline Derivatives as Potential Metallodrugs
Source: Molecules. 2020 May 15;25(10):2322. doi: 10.3390/molecules25102322 (PMC7287719; doi:10.3390/molecules25102322)
Supplement: Supplementary file 1 [file molecules-25-02322-s001.pdf]

Electronic Supplementary Information

Ruthenacarborane–Phenanthroline Derivatives as Potential Metallodrugs

Dr. Martin Kellert [1], Dr. Imola Sárosi [1], Dr. Rajathees Rajaratnam[2], Prof. Dr. Eric Meggers [2], Dr. Peter Lönnecke [1], Prof. Dr. Dr. h.c. Evamarie Hey-Hawkins [1,*]

1 Institute of Inorganic Chemistry, Faculty of Chemistry and Mineralogy, Leipzig University, Johannisallee 29, D-04103 Leipzig, Germany

2 Fachbereich Chemie, Philipps-Universität Marburg, Hans-Meerwein Straße 4, 35043 Marburg, Germany

* Correspondence: hey@uni-leipzig.de; Tel.: +49-341-97-36151 (E. H.-H.)

Table of Content

1. Numbering scheme of all isolated compounds ... 2
2. Additional synthetic procedures for compounds **L1**, **L4** and **1** ... 3
3. Isolation and spectroscopic data of [3-(CO)-3,3-{1',10'-NC_5H_3(5-Br-C_2H)NC_5H_3-κ^2*N,N*}-*closo*-3,1,2-$RuC_2B_9H_{11}$] (**SP1**) ... 5
3.1 Spectroscopic data of **SP1** ... 6
4. Crystallographic data of compounds **2**, **SP1**, **4** and **5** ... 7
5. Protein kinase inhibition ... 10
6. References ... 12

1. Numbering scheme of all isolated compounds

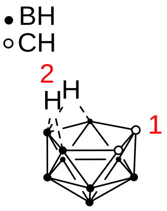

L1

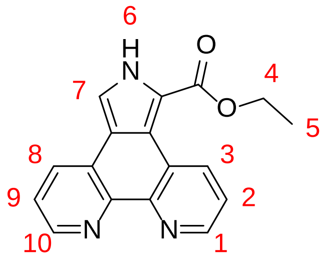

L4'

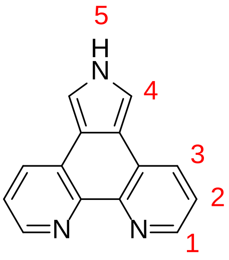

L4

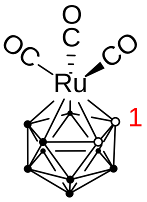

1

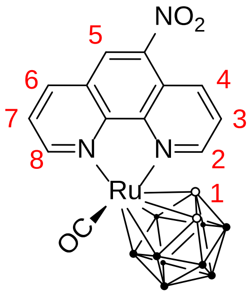

2

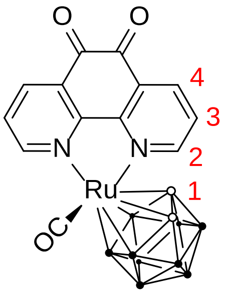

3

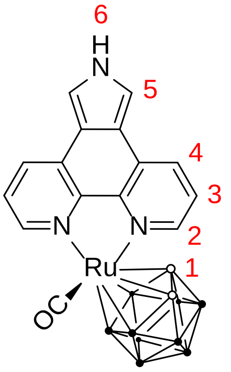

4

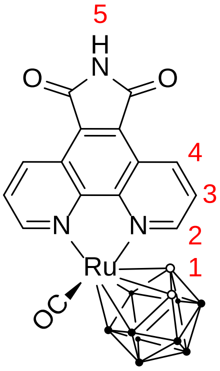

5

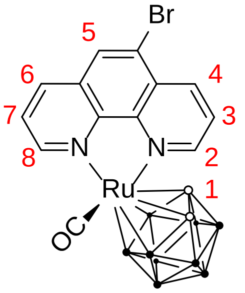

SP1

Figure S1. Numbering scheme of ligands **L1**, **L4’** and **L4** and compounds **1** – **5** and **SP1**.

2. Additional synthetic procedures for compounds L1, L4 and 1

7,8-Dicarba-*nido*-undecaborane(13) (**L1**) [1]: 4.00 g (27.7 mmol, 1.00 eq.) 1,2-dicarba-*closo*-dodecaborane(12) and 3.26 g (58.1 mmol, 2.10 eq.) potassium hydroxide were placed in a 250 mL round bottom flask. The mixture was dissolved in 85 mL methanol and stirred for 30 min at room temperature. Then, the mixture was refluxed for 18 h. After cooling to room temperature, the solvent was removed under reduced pressure. The remaining crude product was treated with 90 mL of benzene and the solvent was removed under reduced pressure and increased temperature until an off-white solid was obtained. The raw product was separated from unreacted 1,2-dicarba-*closo*-dodecaborane(12) by sublimation (10^{-3} mbar, 40 – 50 °C). The resulting colorless residue was dissolved in 65 mL benzene and 21 mL 85% phosphoric acid were added. The mixture was stirred for 17 h at room temperature. Afterwards, the organic layer was separated from the aqueous one. The aqueous layer was extracted two times with 60 mL of diethyl ether. The combined organic layers were dried over sodium sulfate, filtrated and the solvent was removed under reduced pressure. 2.60 g (19.3 mmol, 70%) of **L1** were obtained as a colorless solid. ^{1}H NMR (400 MHz, C_6D_6): δ = −2.36 (s, br, 2 H, 2x H^2), 1.00 to 3.75 (br, 9 H, 9x BH), 2.54 ppm (s, 2 H, 2x CH^1); ^{11}B{^{1}H} NMR (128 MHz, C_6D_6): δ = −27.1 (s, br, 3 B, BH), −16.7 (s, 1 B, BH), −16.1 (s, 1 B, BH), −4.1 (s, 2 B, BH), 4.3 ppm (s, 2 B, BH); ^{11}B NMR (128 MHz, C_6D_6): δ = −27.3 (d, $^{1}J_{BH}$ = 143 Hz, 1 B, BH), −26.9 (d, $^{1}J_{BH}$ = 145 Hz, 2 B, BH), −16.4 (m, br, 2 B, BH), −4.1 (d, $^{1}J_{BH}$ = 152 Hz, 2 B, BH), 4.4 ppm (d, $^{1}J_{BH}$ = 163 Hz, 2 B, BH).

1,10-Phenanthrolinopyrrole (**L4**) [2]: 2.24 g (9.95 mmol, 1.00 eq.) 5-nitro-1,10-phenanthroline (**L2**) were placed in a 250 mL round bottom flask and dissolved in 120 mL tetrahydrofuran. The mixture was stirred for 40 min at room temperature. To this mixture, first, 1.18 mL (1.22 g, 10.8 mmol, 1.09 eq.) ethyl isocyanoacetate and then 3.00 mL (3.06 g, 20.1 mmol, 2.02 eq.) 1,8-diazabicyclo[5.4.0]undec-7-ene (DBU) were added. The reaction mixture was allowed to stir for 20 h at room temperature. After the reaction was finished, half of the solvent was removed under reduced pressure. The resulting yellow precipitate, intermediate **L4’**, was filtered off, washed three times with 10 mL diethyl ether and dried in vacuum. 1,10-Phenanthrolinopyrrole ethyl ester (**L4’**): ^{1}H NMR (400 MHz, DMSO-*d*$_6$): δ = 1.44 (t, $^{3}J_{HH}$ = 7.1 Hz, 3 H, $CH_3{}^5$), 4.45 (q, $^{3}J_{HH}$ = 7.1 Hz, 2 H, $CH_2{}^4$), 7.72 (m, 2 H, CH^2 and CH^9), 8.41 (s, 1 H, CH^7), 8.82 (dd, $^{3}J_{HH}$ = 8.0 Hz, $^{4}J_{HH}$ = 1.5 Hz, 1 H, CH^{10}), 8.90 (dd, $^{3}J_{HH}$ = 4.3 Hz, $^{4}J_{HH}$ = 1.5 Hz, 1 H, CH^8), 8.96 (dd, $^{3}J_{HH}$ = 4.2 Hz, $^{4}J_{HH}$ = 1.6 Hz, 1 H, CH^3), 10.10 ppm (dd, $^{3}J_{HH}$ = 8.3 Hz, $^{4}J_{HH}$ = 1.6 Hz, 1 H, CH^1).

The yellow solid (**L4'**) was placed in a 500 mL round bottom flask and suspended in 150 mL of a 1:1 mixture of ethanol and 0.2 M sodium hydroxide solution. The reaction mixture was heated under reflux for 8 h. After cooling to room temperature, stirring was continued for 30 min. The resulting precipitate was filtered off, washed three times with 12 mL diethyl ether and was dried in vacuum. 1.18 g (5.37 mmol, 54%) of the title compound **L4** were obtained as a beige powder. **L4**: ^{1}H NMR (400 MHz, DMSO-d_6): δ = 7.61 (m, 2 H, 2x CH^2), 7.96 (s, 2 H, 2x CH^4), 8.61 (m, 2 H, 2x CH^3), 8.77 (m, 2 H, 2x CH^1), 12.22 ppm (s, br, 1 H, NH^5); IR (KBr): $\tilde{\nu}$ = 3185 (s, νCH-sp^2), 3085 (s, νCH-sp^2), 1638 (m, νCN-sp^2), 1582 (m, νCC-sp^2), 1557 (s, νCC-sp^2), 1501 (w, δNH), 801 cm^{-1} (m, δCH); MS (ESI, pos.): found: m/z (%): 220 (100) $[M+H]^+$, 242 (78) $[M+Na]^+$; calcd: m/z: 220 $[M+H]^+$, 242 $[M+Na]^+$.

[3-$(CO)_3$-*closo*-3,1,2-$RuC_2B_9H_{11}$] (**1**) [3]: 3.90 g (6.10 mmol, 0.33 eq.) triruthenium dodecacarbonyl were placed in a 250 mL round bottom flask. Then a solution of 2.50 g (18.6 mmol, 1.00 eq.) 7,8-dicarba-*nido*-undecaborane(13) (**L1**) in 60 mL cyclohexane (in the literature, *n*-heptane is used) was added. The mixture was heated under reflux for 7 h. After the reaction was finished, half of the solvent was removed under reduced pressure. The resulting precipitate was filtered off and washed twice with a 2:1 mixture of dichloromethane and petroleum ether (bp. 40 – 60 °C). The combined organic layers were filtered through a celite pad (3 cm) and the filtrate was dried under vacuum. The resulting residue was washed two times with 10 mL petroleum ether (bp. 40 – 60 °C). Finally, the raw product was purified by column chromatography (dichloromethane/*n*-hexane, 1:1, (v/v); R_f = 0.51). 3.56 g (11.2 mmol, 60%) of the title compound **1** were obtained as a dark red solid. ^{1}H NMR (400 MHz, $CDCl_3$): δ = 1.40 to 4.60 (br, 9 H, 9x BH), 3.48 (s, 2 H, 2x CH^1); $^{11}B\{^1H\}$ NMR (128 MHz, $CDCl_3$): δ = −17.4 (s, br, 3 B, BH), −8.6 (s, br, 2 B, BH), −4.3 (s, 2 B, BH), −3.1 (s, 1 B, BH), 9.6 ppm (s, 1 B, BH); ^{11}B NMR (128 MHz, $CDCl_3$): δ = −17.4 (d, br, $^1J_{BH}$ = 160 Hz, 3 B, BH), −8.6 (d, br, $^1J_{BH}$ = 152 Hz, 2 B, BH), −3.7 (m, br, 3 B, BH), 9.7 ppm (d, $^1J_{BH}$ = 148 Hz, 1 B, BH); IR (KBr): $\tilde{\nu}$ = 2566 (m, νBH-sp^3), 2118 (s, νCO-sp), 2056 cm^{-1} (s, νCO-sp); elemental analysis (%): ($RuC_5H_{11}B_9O_3$) found: C 18.70, H 3.14; calcd: C 18.91, H 3.49. C, H analysis was conducted with an elemental analyzer Vario EL from Heraeus.

3. Isolation and spectroscopic data of [3-(CO)-3,3-{1’,10’-NC_5H_3(5-Br-C_2H)NC_5H_3-κ²*N,N*}-*closo*-3,1,2-$RuC_2B_9H_{11}$] (SP1)

The synthesis of [3-(CO)-3,3-{1’,10’-NC_5H_3(5-Br-C_2H)NC_5H_3-κ²*N,N*}-*closo*-3,1,2-$RuC_2B_9H_{11}$] (**SP1**) was performed in the same way like for compound **2**. After column chromatography two fractions were isolated. The second fraction was the desired product **3** with an R_f value of 0.17 in DCM and a yield of 73%. The first fraction turned out to be [3-(CO)-3,3-{1’,10’-NC_5H_3(5-Br-C_2H)NC_5H_3-κ²*N*,*N*}-*closo*-3,1,2-$RuC_2B_9H_{11}$] (**SP1**) with an R_f value of 0.78 in DCM and a yield of 27% (the spectroscopic data of **SP1** are reported at the end of this paragraph). Since **L3** was synthesized following the procedure of Sergeeva *et al.* it is possible to explain the formation of 5-bromo-1,10-phenanthroline (**L5**) [4,5]. As reagents for this synthesis, potassium bromide and a mixture of concentrated sulfuric acid and concentrated nitric acid were used. During this reaction, the bromide is oxidized to bromine which adds to the aromatic system to form **L5** which is then further oxidized to the desired ligand **L3**. Indeed, the bromo derivative is a possible side product in this reaction. Following the given procedure, there is no extra purification step, which means **L5** turns out to be a possible impurity. Later, NMR studies of **L3** showed that **L5** is in fact the assumed impurity in this ligand fraction.

Side product **SP1** was characterized with the commonly used spectroscopic methods. Furthermore, orange plates of **SP1** suitable for X-ray structure determination were obtained. The molecular structure is shown in Figure S2. **SP1** crystallizes in the monoclinic space group $P2_1/n$ with one molecule dichloromethane in the asymmetric unit. It is a chiral molecule, with ruthenium as the chiral center. Although the wR_2 value of 13% is not good, the identity of **SP1** is confirmed.

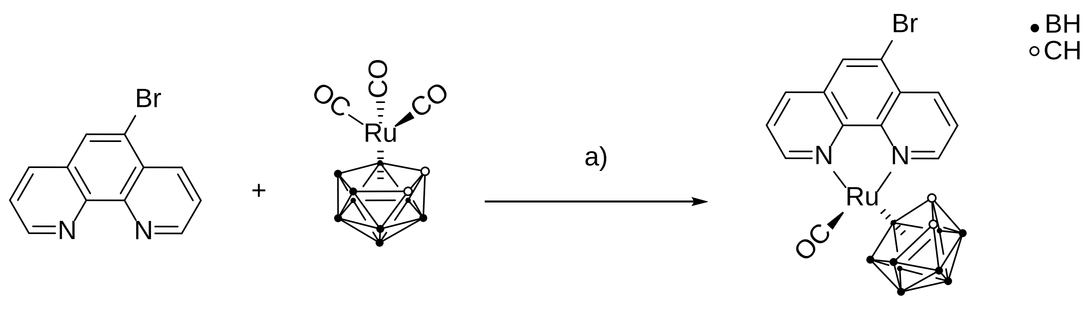

L5 **1** **SP1**

Scheme S1. Formation reaction of [3-(CO)-3,3-{1’,10’-NC_5H_3(5-Br-C_2H)NC_5H_3-κ²*N*,*N*}-*closo*-3,1,2-$RuC_2B_9H_{11}$] (**SP1**) as a side product. a) MeCN, trimethylamine *N*-oxide, rt, 84 h, 27%.

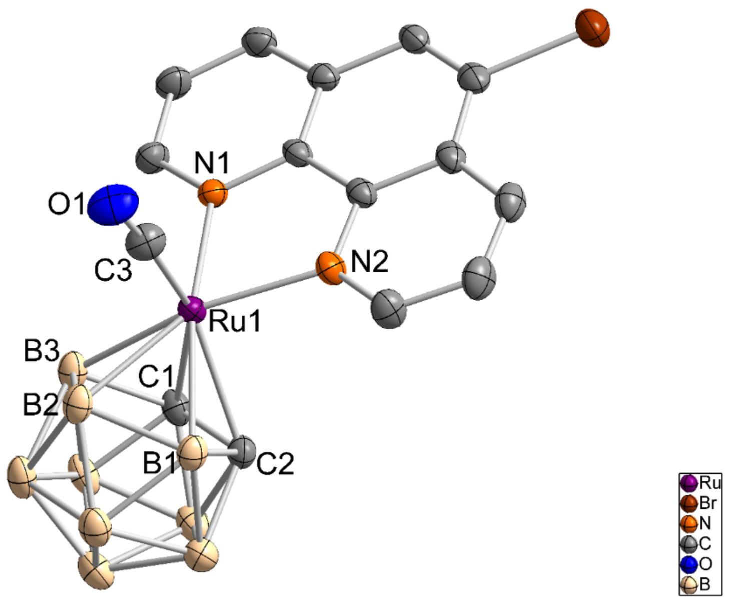

Figure S2. Molecule structure of [3-(CO)-3,3-{1',10'-NC_5H_3(5-Br-C_2H)NC_5H_3-κ^2*N*,*N*}-*closo*-3,1,2-$RuC_2B_9H_{11}$] (**SP1**) (only the *S* enantiomer is shown) as an ellipsoid-stick model with thermal ellipsoids at 50% probability level. Solvent molecules, hydrogen atoms and the 12% disordered part of the molecule are omitted for clarity.

3.1 Spectroscopic data of SP1

^{1}H NMR (400 MHz, CD_3CN): δ = 0.75 to 2.80 (br, 9 H, BH), 3.36 (s, br, 2 H, 2x CH^1), 7.93 (dd, $^3J_{HH}$ = 8.2 Hz, $^4J_{HH}$ = 5.2 Hz, 1 H, CH^7), 8.02 (dd, $^3J_{HH}$ = 8.4 Hz, $^4J_{HH}$ = 5.3 Hz, 1 H, CH^3), 8.55 (s, 1 H, CH^5), 8.63 (m, 1 H, CH^6), 8.92 (m, 1 H, CH^4), 9.38 (m, 1 H, CH^8), 9.42 ppm (m, 1 H, CH^2).

^{11}B{^{1}H} NMR (128 MHz, CD_3CN): δ = –22.2 (s, br, 1 B, BH), –21.4 (s, 2 B, BH), –9.8 (s, 2 B, BH), –8.8 (s, 2 B, BH), –6.9 (s, 1 B, BH), –2.1 ppm (s, br, 1 B, BH).

^{11}B NMR (128 MHz, CD_3CN): δ = –21.9 (m, br, 3 B, BH), –9.3 (m, br, 4 B, BH), –6.8 (d, $^1J_{BH}$ = 133 Hz, 1 B, BH), –2.0 ppm (d, $^1J_{BH}$ = 129 Hz, 1 B, BH).

4. Crystallographic data of compounds 2, SP1, 4 and 5

The data were collected on a Gemini diffractometer (Rigaku Oxford Diffraction) using Mo-K_α radiation (λ = 71.073 pm), ω-scan rotation. Data reduction was performed with CrysAlis Pro [6a] including the program SCALE3 ABSPACK for empirical absorption correction. The structures were solved by direct methods with SHELXS-2013 or -2014 [6b] (**2**, **SP1**, **4**) or SIR92 (**5**) [6c] and the refinement of all non-hydrogen atoms was performed with SHELXL-2018 [6a]. With the exception of some disordered parts of the structure all non-hydrogen atoms were refined with anisotropic displacement parameters. All carborane carbon atoms could be localized with a bond length and displacement parameter analysis. For one of the two molecules in **4**, these carbon atoms (C18, C19) are slightly disordered. CCDC 1993591 (**2**), 1993592 (**SP1**), 1993593 (**4**) and 1993594 (**5**) contain the supplementary crystallographic data for this paper. These data can be obtained free of charge via https://summary.ccdc.cam.ac.uk/structure-summary-form (or from the Cambridge Crystallographic Data Centre, 12 Union Road, Cambridge CB2 1EZ, UK; fax: (+44)1223-336-033; or deposit@ccdc.cam.uk).

Table S1. Fundamental crystallographic data of compounds **2**, **SP1**, **4** and **5**.

**Compound**	**2**†	**SP1***	**4**	**5**
Empirical formula	$C_{16}H_{20}B_9Cl_2N_3O_3Ru$	$C_{16}H_{20}B_9BrCl_2N_2ORu$	$C_{17}H_{20}B_9N_3ORu$	$C_{23}H_{27}B_9N_6O_3Ru$
Formula weight	571.61	605.51	480.72	633.86
Temperature [K]	130(2)	130(2)	130(2)	170(2)‡
Crystal system	Monoclinic	Monoclinic	Triclinic	Triclinic
Space group	$P2_1/n$	$P2_1/n$	$P\bar{1}$	$P\bar{1}$
Unit cell dimensions				
a [pm]	1100.0(1)	1098.33(2)	1103.27(4)	1031.29(4)
b [pm]	1815.6(1)	1898.24(2)	1367.75(6)	1151.40(4)
c [pm]	1222.3(1)	1149.62(2)	1559.03(7)	1218.38(3)
α [°]	90	90	95.329(4)	85.980(2)
β [°]	111.62(1)	109.438(2)	108.094(4)	84.258(2)
γ [°]	90	90	112.291(4)	81.377(3)
Volume [nm^3]	2.2693(4)	2.26022(7)	2.0089(2)	1.42097(8)
Z	4	4	4	2
$\rho_{(calculated)}$ [Mg/m^3]	1.673	1.779	1.589	1.481
μ [mm^{-1}]	0.953	2.712	0.796	0.591

F(000)	1136	1184	960	640
Crystal size [mm^3]	0.27 · 0.10 · 0.01	0.3 · 0.2 · 0.15	0.2 · 0.1 · 0.01	0.25 · 0.25 · 0.2
Θ_{Min} / Θ_{Max} [°]	2.114 / 23.257	2.163 / 33.141	2.831 / 26.369	2.388 / 32.705
Index ranges	–12 ≤ h ≤ 12<br>–20 ≤ k ≤ 20<br>–13 ≤ l ≤ 13	–16 ≤ h ≤ 15<br>–29 ≤ k ≤ 29<br>–17 ≤ l ≤ 17	–13 ≤ h ≤ 13<br>–17 ≤ k ≤ 17<br>–19 ≤ l ≤ 19	–15 ≤ h ≤ 15<br>–17 ≤ k ≤ 16<br>–17 ≤ l ≤ 18
Reflections collected	3886	37484	19273	21657
Indep. refl. [R_{int}]	3886 [0.1869]	8609 [0.0338]	8205 [0.0428]	9362 [0.0286]
Completeness; (θ [°])	99.8 %; (23.26)	100.0 %; (33.14)	99.9 %; (26.37)	100.0 %; (30.51)
Absorption correction	Semi-empirical from equivalents	Semi-empirical from equivalents	Semi-empirical from equivalents	Semi-empirical from equivalents
T_{Max} / T_{Min}	1 / 0.77574	1 / 0.84265	1 / 0.99345	1 / 0.98991
Refinement method	Full-matrix least-squares on F^2	Full-matrix least-squares on F^2	Full-matrix least-squares on F^2	Full-matrix least-squares on F^2
Restraints / parameters	312 / 312	59 / 354	0 / 648	3 / 450
Goodness-of-fit on F^2	0.745	1.048	1.010	1.037
R_1, wR_2 [I>2σ(I)]	0.0576, 0.0843	0.0480, 0.1201	0.0389, 0.0706	0.0332, 0.0738
R_1, wR_2 (all data)	0.1779, 0.0986	0.0640, 0.1300	0.0653, 0.0782	0.0408, 0.0776
Residual electron density [e·Å^{-3}]	0.783 / –0.476	1.960 / –1.489	0.476 / –0.577	0.731 / –0.544

† Twinned crystal

‡ The crystals tend to crack at temperatures below 170 K

* As a result of one disordered bromine substituent, the whole complex is slightly disordered with a ratio of 0.885(2):0.115(2). The minor 12% disordered part was only calculated for ruthenium and the phenanthroline unit.

Table S2. Selected bond lengths (in pm) and bond angles (in °) of **SP1** compared to [3-CO-3,3-(bipy-κ^2*N,N*)-*closo*-3,1,2-$RuC_2B_9H_{11}$] (**I**) (bipy = 2,2'-bipyridine) [7].

Atom group	SP1	I
Ru(1)–C(1)	226.8(3)	217.4(4)
Ru(1)–C(2)	227.6(3)	222.4(4)
Ru(1)–B(1)	225.8(4)	227.9(4)
Ru(1)–B(2)	224.2(3)	227.7(4)
Ru(1)–B(3)	223.1(4)	220.6(5)
Ru(1)–C(3)	183.4(3)	186.6(4)
Ru(1)–N(1)	213.7(4)	209.3(3)
Ru(1)–N(2)	213.5(5)	213.5(3)
N(1)···N(2)	263.3(6)	261.0(5)
C(3)–O(1)	115.0(4)	115.4(5)
N(1)–Ru(1)–C(3)	95.0(1)	90.1(2)
N(2)–Ru(1)–C(3)	92.9(2)	92.0(1)
N(1)–Ru(1)–N(2)	76.1(1)	76.3(1)
Ru(1)–C(3)–O(1)	175.0(3)	175.3(4)

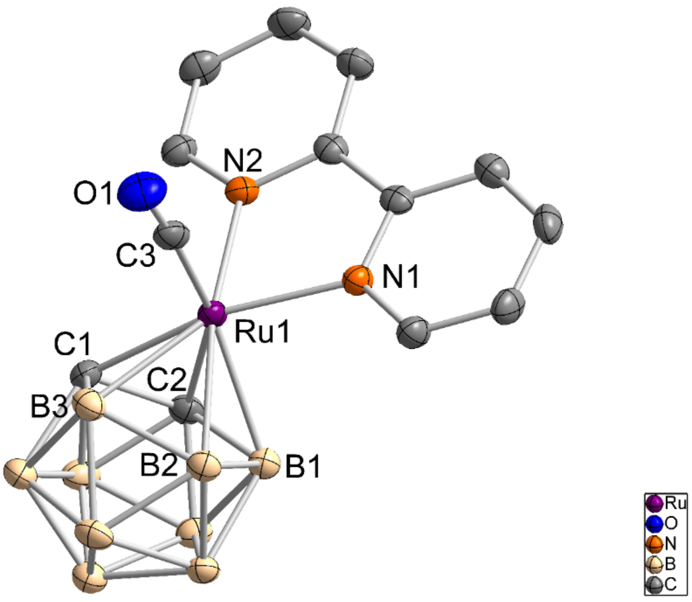

Figure S3. Molecule structure of [3-CO-3,3-(bipy-κ^2*N,N*)-*closo*-3,1,2-$RuC_2B_9H_{11}$] (**I**) (bipy = 2,2'-bipyridine) as an ellipsoid-stick model with thermal ellipsoids at 50% probability level. Solvent molecules and hydrogen atoms are omitted for clarity [7].

5. Protein kinase inhibition

Preparation of buffer solutions for the protein kinase assays:

Reaction buffer 1 (RB1): RB1 was prepared with a final concentration of 40 mM 3-(*N*-morpholino)propansulfonic acid/sodium hydroxide, and 50 mM magnesium acetate at a pH of 7.0, aliquoted and stored at –20 °C until use.
Reaction buffer 2 (RB2): RB2 was prepared with a final concentration of 20 mM 3-(*N*-morpholino)propanesulfonic acid/sodium hydroxide, 1 mM ethylenediaminetetraacetic acid (EDTA), 0.01% Brij 35, 5% 2-mercaptoethanol, and 1 mg/mL bovine serum albumin (BSA) at pH 7.0, aliquoted and stored at –20 °C until use.

Pim1 aliquots: A stock solution of 10 µg of Pim-1 in 8.4 µL buffer, purchased from Merck Millipore, was diluted by adding 22.8 µL of RB2. This stock solution was aliquoted in 2 µL portions which were stored at –70 °C until use.
Pim1 substrate S6 aliquots: 1 mg of the Pim-1 kinase substrate S6 (H-Lys-Lys-Arg-Asn-Arg-Thr-Leu-Thr-Val-OH, Anaspec, Cat.No.: 63865, Lot.: 68951) was diluted using RB1 to a concentration of 500 µM, aliquoted, and stored at –70 °C until use.

Protein kinase inhibition experiments: The prepared aliquots of Pim-1 and the kinase substrate S6 were thawed on the day of experiment on ice up to 4 °C. The Pim-1 kinase was further diluted by adding 98 µL of RB2 to a total of a 100 µL working solution at 4 °C. 5 µL of RB1, 2.5 µL of the kinase substrate S6 stock solution, 7.5 µL of purified water, 2.5 µL of Pim-1 working solution, and 2.5 µL of the complex **4** or **5** as DMSO stock solutions were preincubated for 30 min at ambient temperature. The reaction was then initiated by adding ATP in a final concentration of 1 µM and approximately 0.1 µCi/µL of [$^{\gamma\text{-}33}$P]-ATP. Reactions were performed in a total volume of 25 µL. After an incubation time of 45 min at ambient temperature, the reaction was terminated by spotting 17.5 µL of the reaction mixture on a circular P81 phosphocellulose paper (2.1 cm diameter, Whatman), followed by washing three times for 5 min with 0.75% phosphoric acid and once with acetone. The dried P81 papers were transferred to scintillation vials and added with 5 mL of scintillation cocktail (purchased from Roth). The counts per minute (CPM) were measured using a Beckmann Coulter LS6500 multipurpose scintillation counter and corrected by the background CPM. The IC_{50} values were determined in duplicate for each single concentration. The experiments were repeated independently under the same conditions to verify the results. Nonlinear regression and data evaluation were performed using OriginPro 8G (OriginLab).

Results: The IC_{50} determinations show a low reproducibility which we trace back to the high hydrophobicity of the compounds. IC_{50} values of compounds **4** and **5** are in the range of 50 – 200 μM with complex **5** showing a slightly higher potency.

6. References

1. Hlatky, G.G.; Crowther, D.J. Main Group and Transition Metal Cluster Compounds: 38. 7,8-Dicarbaundecaborane(13). *Inorg. Syn.* **1998**, *32*, 229–231.

2. Villegas, J.M.; Stoyanov, S.R.; Rillema, D.P. Synthesis and Photochemistry of Ru(II) Complexes Containing Phenanthroline-Based Ligands with Fused Pyrrole Rings. *Inorg. Chem.* **2002**, *41*, 6688–6694, doi:10.1021/ic020436a.

3. Anderson, S.; Mullica, D.F.; Sappenfield, E.L.; Stone, F.G.A. Carborane Complexes of Ruthenium: A Convenient Synthesis of [$Ru(CO)_3(\eta^5$-7,8-$C_2B_9H_{11})$] and a Study of Reactions of This Complex. *Organometallics* **1995**, *14*, 3516–3526.

4. Wang, C.; Lystrom, L.; Yin, H.; Hetu, M.; Kilina, S.; McFarland, S.A.; Sun, W. Increasing the Triplet Lifetime and Extending the Ground-state Absorption of Biscyclometalated Ir(III) Complexes for Reverse Saturable Absorption and Photodynamic Therapy Applications. *Dalton Trans.* **2016**, *45*, 16366–16378, doi:10.1039/c6dt02416e.

5. Sergeeva, N.N.; Donnier-Marechal, M.; Vaz, G.M.; Davies, A.M.; Senge, M.O. Stability and Spectral Properties of Europium and Zinc Phenanthroline Complexes as Luminescent Probes in High Content Cell-Imaging Analysis. *J. Inorg. Biochem.* **2011**, *105*, 1589–1595.

6. a) Rigaku Oxford Diffraction, (1995–2020), CrysAlisPro Software system, Rigaku Corporation, Oxford, UK.

   b) Sheldrick, G.M. SHELX-20xy including SHELXS and SHELXL. *Acta Crystallogr.* **2015**, *C71*, 3–8.

   c) Altomare, A.; Cascarano, G.; Giacovazzo, C.; Guagliardi, A. SIR92. *J. Appl. Crystallogr.* **1993**, *26*, 343-350.

7. Jelliss, P.A.; Mason, J.; Nazzoli, J.M.; Orlando, J.H.; Vinson, A.; Rath, N.P.; Shaw, M.J. Synthesis and Characterization of Ruthenacarborane Complexes Incorporating Chelating N-Donor Ligands: Unexpected Luminescence From the Complex 3-CO-3,3-κ^2-$Me_2N(CH_2)_2NMe_2$-*closo*-3,1,2-$RuC_2B_9H_{11}$. *Inorg. Chem.* **2006**, *45*, 370–385, doi:10.1021/ic051463e.
